# Impact of an evidence-based order panel on antibiotic prescribing in ambulatory patients with cystitis

**DOI:** 10.1017/ash.2025.62

**Published:** 2025-04-21

**Authors:** Matt Neumann, Ryan W. Stevens, Kristin Cole, Paschalis Vergidis, Abinash Virk, Dan Ilges, Kelsey L. Jensen

**Affiliations:** 1 Department of Pharmacy, Mayo Clinic Health System, Austin, MN, USA; 2 Department of Pharmacy, Mayo Clinic, Rochester, MN, USA; 3 Division of Clinical Trials and Biostatistics, Mayo Clinic, Rochester, MN, USA; 4 Division of Public Health, Infectious Diseases, and Occupational Medicine, Mayo Clinic, Rochester, MN, USA; 5 Department of Pharmacy, Mayo Clinic Arizona, Phoenix, AZ, USA

## Abstract

**Background:**

Optimizing antibiotic prescribing for urinary tract infections (UTI) represents an opportunity for ambulatory antibiotic stewardship programs (ASPs). A pre-populated order panel for UTI was implemented in the Mayo Clinic Enterprise in May 2022. The order panel provides antibiotic regimens aligning with institutional guidelines according to patient characteristics, presence or absence of complicating features, and antimicrobial allergy status. We assessed impacts of panel use on prescribing practices for cystitis.

**Methods:**

This retrospective cohort study of ambulatory encounters with a primary diagnosis of cystitis from May 16, 2022, to May 15, 2023, compared encounters in which the order panel was utilized to encounters managed without the panel. The primary outcome was concordance with institutional guidelines, including drug selection, dose/frequency, and duration. Secondary outcomes included rate of repeat healthcare contact for UTI within 14 days and total duration of therapy.

**Results:**

793 randomly selected patient encounters (397 panel and 396 non-panel) were included. Prescribing was guideline adherent in 79.3% and 64.9% (*P* < 0.001) of panel and non-panel encounters, respectively. There were more 3- and 5-day treatment courses in the panel cohort; however, inappropriate duration of therapy was the most common reason for non-concordance in both cohorts. There was no significant difference between groups in repeat 14-day healthcare contact for UTI (13.4% panel vs 11.1% no panel, *P* = 0.34).

**Conclusion:**

Use of a pre-populated ambulatory order panel for the treatment of cystitis was associated with greater concordance with institutional guidelines, without adversely impacting repeat healthcare contact for UTI.

## Introduction

Over 230 million outpatient antibiotic prescriptions were written in the United States in 2022, with estimates of 30-50% being inappropriate or unnecessary.^1,2^ Antibiotic overuse can contribute to increases in antibiotic resistance, morbidity, and financial burden on the healthcare system.^3^ In recent years, The Joint Commission (TJC) has placed an emphasis on ambulatory ASP, issuing standard *MM.09.01.03* requiring implementation of ASPs for institutions with ambulatory TJC accreditation.^4^ These standards include identification of local leaders or practice champions, providing education for best practices, tracking ambulatory antibiotic use metrics, and implementation of interventions to improve concordance with practice guidelines.

Urinary tract infections (UTI) are one of the most common infectious indications for ambulatory patient encounters, with one study identifying that 8.1% of all urgent care visits were attributed to UTI, exceeded by only respiratory tract (41.8%) and skin and soft tissue infections (13.7%). Yet, the rate of antimicrobial prescribing in UTI encounters was found to be 76%, compared to 50% and 35% for respiratory and skin and soft tissue infection encounters, respectively.^5^ It is estimated that 60% of women will experience at least one UTI in their lifetime with about 30% experiencing at least one recurrence within six months of the index infection.^6^ In the absence of complicating factors (eg, male sex, pregnancy, poorly controlled diabetes, urinary obstruction, symptoms for greater than 1 week), current guidelines recommend antibiotic courses as short as 3–5 days for women, depending on the agent selected.^7^

Antimicrobial prescribing concordance with national guidelines for treatment of UTI is often low, with many studies demonstrating clear opportunity for optimizing antibiotic appropriateness.^8–10^ One study found that only about 30% of prescriptions for UTI were optimally prescribed.^11^ Among prescriptions identified as inappropriate, common features lending to inappropriateness included prolonged durations of therapy and overuse of non-preferred antimicrobials (including fluoroquinolones). Potential drivers of non-concordance include diagnostic uncertainty, fear of treatment failure, and patient expectations.^12–14^

As one of several tactics to optimize ambulatory antibiotic prescribing in UTIs within the Mayo Enterprise, an order panel within the electronic health record (EHR) was implemented on May 16^th^, 2022. This syndromic panel included clinical decision support (CDS) functionality similar to a treatment panel previously developed to optimize prescribing in upper respiratory infections.^15^ The UTI order panel (**Supplement** 1) consists of pre-populated, guideline-adherent laboratory testing and antibiotic orders, including agent selection, dose, and duration of therapy, for asymptomatic bacteriuria (ASB), uncomplicated cystitis, complicated cystitis, and pyelonephritis. The panel was promoted to providers Enterprise-wide through internal communications, and via departmental presentations on the regional and local levels. The objective of this study is to evaluate impact of order panel utilization on the concordance of antibiotic prescribing with institutional practice guidelines in uncomplicated and complicated cystitis in adult patients.

## Methods

We conducted a retrospective review of Mayo Enterprise ambulatory encounters for cystitis from panel implementation on May 16, 2022, to May 15, 2023. The Mayo Enterprise includes three major centers in Minnesota, Florida, and Arizona, and the Mayo Clinic Health System, a network of hospitals and clinics in Minnesota and Wisconsin. Patients were included if they were at least 18 years of age, seen in primary care or urgent care, prescribed an antibiotic used in the treatment of cystitis, and had an ICD-10 code(s) consistent with lower urinary tract infection (**Supplement** 2) as the primary diagnosis code for the encounter. Patients were excluded if they had a diagnoses code consistent with pyelonephritis, ASB, UTI not otherwise specified, symptoms of flank pain or costovertebral angle tenderness documented in clinical notation, had indwelling ureteral stent, ileal conduit, nephrostomy tube, isolation of a urinary pathogen in previous 90-days, urinary instrumentation within previous 30-days, active antibiotic at time of encounter, antibiotics prescribed for an infectious indication other than cystitis, or antibiotics prescribed for greater than 28-days at the index encounter. Given the outpatient nature of the study, infrequency of patient-initiated encounters for ASB, and rarity of antimicrobial prescribing for this condition in this care setting, patients with ASB were excluded. Pyelonephritis was excluded given differences in both recommended durations of therapy (ie 7–10 d for pyelonephritis vs 5–7 d for cystitis) and preferred antibiotic selection as compared with cystitis. For patients treated in Minnesota, those without Minnesota Research Authorization were excluded. For patients with multiple UTI encounters during the study timeframe, only the first encounter was included.

Data was extracted from the EHR using Epic SlicerDicer (Epic, Verona, WI) and manual chart abstraction. Data included encounter characteristics, patient demographics, diagnoses, laboratory and microbiological data, allergies, UTI complicating factors, prescribing characteristics, documented adverse effects, and repeat healthcare contact for UTI-related indications. Encounter characteristics collected included primary encounter diagnosis and symptom ICD-10 codes, encounter type (ie, in-person, telemedicine, and non-visit care), and provider type (ie, physician, advanced practice practitioner (APP), other). We defined complicated cystitis as patients meeting one or more of the following criteria: male sex, age greater than 65, pregnant, symptom duration greater than 7 days, recent antimicrobial use (within the last 30 d), poorly controlled diabetes (A1c >7%), history of infection with multidrug-resistant organism(s), urinary obstruction or anatomic abnormality of urinary tract, current indwelling ureteral stent, nephrostomy tube, urinary diversion, or renal transplant.

The primary outcome was institutional guideline concordance (**Supplement**). An encounter had to demonstrate appropriate antibiotic selection, dose/frequency, and duration to be considered concordant. Institutional guidelines within the Mayo Clinic Enterprise are based on national guidelines, developed via Enterprise-wide expert consensus, and incorporate evaluations of antibiogram data from all regions. Secondary outcomes evaluated include rate of any repeat healthcare contact for a UTI indication within 14 days of completion of the antibiotic regimen prescribed at the index encounter, which was identified through manual chart review, utilization rates of individual antibiotics, antibiotic changes, and prescribed durations of therapy.

### Statistical analysis

Data was summarized using frequencies and percentages for categorical data, and either means and standard deviations or medians and interquartile ranges (IQR) for continuous data. Patient and encounter characteristics were compared between the panel and non-panel groups using either a Chi-square or Fisher’s exact test for categorical data, and either a t-test or Wilcoxon rank sum test for continuous data. Multivariable logistic regression was used to assess the association between panel use and guideline-concordant prescribing after adjusting for age, sex, encounter type, primary provider type, beta-lactam allergy, sulfamethoxazole/trimethoprim allergy, CrCl, complicated cystitis, symptom duration >7 days, and recent antibiotic use. These features were determined a priori based on study team’s hypothesis of factors likely to influence prescribing concordance rates. Concerns for collinearity were low given variance inflation factors below 5. A C-statistic of 0.84 was calculated, indicating a strong model. Associations were summarized using odds ratios (OR) and 95% confidence intervals (CI). All analyses were performed using SAS version 9.4 software (SAS Institute, Inc.; Cary, NC), and p-values ≤ 0.05 were considered statistically significant. Based on an estimated concordance rate of 50% from other infectious syndromes previously evaluated; by enrolling the same number of patients in each group we needed 794 patients total (397 in each group) to achieve 80% power to detect a difference of 10% or more between groups. Encounters meeting initial inclusion criteria were randomized, and cohorts were collected up to the aforementioned power threshold.

## Results

**Figure 1.**
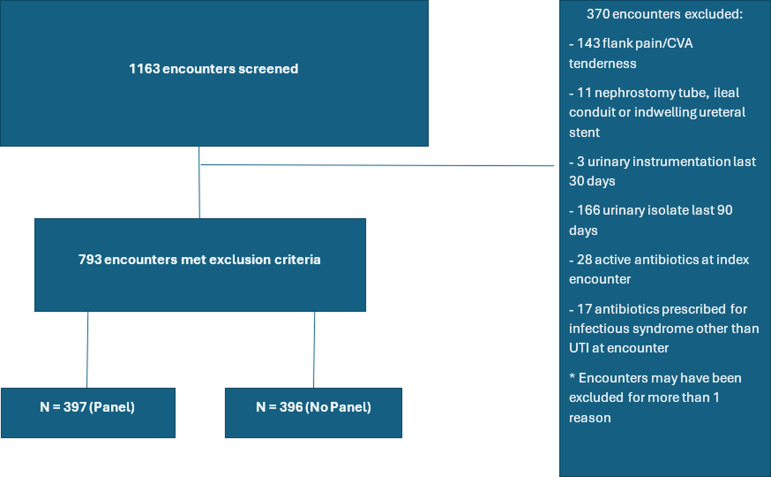
Encounter enrollment.

A total of 14,085 encounters met initial inclusion criteria across the enterprise, with panel use observed in 1,220 (8.7%) encounters as compared with nonuse in 12,865 (91.3%). A total of 1,163 encounters were screened to enroll 793 randomly selected patients in the analysis, representing 397 in the panel and 396 in the non-panel cohorts (Figure 1). There were no significant differences in baseline characteristics between groups (Table 1). A statistically significant difference was observed in provider type between the panel and non-panel cohorts, with panel use being more common amongst encounters conducted by APPs (*P* = 0.004). For the entire cohort, the majority were female (94.8%) and Caucasian (94.7%), with mean age of 55 years. Most presented with urinary symptoms including dysuria (85%), frequency (78.6%), and urgency (58.4%). Urinary cultures were ordered in 62.3% of patients. Of those patients, microbial growth was observed in 84% of encounters, with the organism reported as sensitive to empiric treatment in 90.1% of all encounters (Table 1).

**Table 1.** Baseline characteristics

Characteristic	Panel (N = 397)	No Panel (N = 396)	Total (N = 793)	P-Value
**Sex (Female)**	376 (94.7)	376 (94.9)	752 (94.8)	0.88
**Age at Encounter (Mean)**	55.5 (22.0)	54.0 (21.0)	54.8 (21.5)	0.33
**Height (cm)**	164.5 (7.0)	164.7 (8.3)	164.6 (7.6)	0.78
**Weight (kg)**	83.0 (22.5)	80.4 (21.3)	81.7 (21.9)	0.13
**Listed Allergy** **Beta-Lactam** **SMX/TMP**	75 (18.9) 74 (18.6)	77 (19.4) 68 (17.2)	152 (19.2) 142 (17.9)	0.84 0.59
**Pregnancy**	1 (0.3)	4 (1.0)	5 (0.6)	0.18
**Race** **Caucasian** **Black/AA** **AI/Alaskan Native** **Hispanic/Latino** **Asian** **Not Listed/Refused**	381 (96) 6 (1.5) 1 (0.3) 10 (2.5) 5 (1.3) 5 (1.3)	370 (93.4) 7 (1.8) 4 (1.0) 9 (2.3) 5 (1.3) 8 (2)	751 (94.7)13 (1.6) 5 (0.6) 19 (2.4) 10 (1.3) 13 (1.6)	0.11 0.78 0.18 0.82 >0.99 0.4
**Encounter Type** **In-Person** **Telemedicine** **Non-Visit Care**	273 (68.8) 74 (18.6) 50 (12.6)	258 (65.2) 88 (22.2) 50 (12.6)	531 (67.0) 162 (20.4) 100 (12.6)	0.44
**Primary Provider Type** **APP** **MD/DO** **Other**	313 (78.8) 81 (20.4) 3 (0.8)	271 (68.4) 120 (30.3) 5 (1.3)	584 (73.6) 201 (25.3) 8 (1.0)	0.004
**Primary Diagnosis** **Cystitis** **Dysuria** **Frequency** **Urgency**	232 (58.4) 131 (33.0) 27 (6.8) 7 (1.8)	167 (42.2) 193 (48.7) 31 (7.8) 5 (1.3)	399 (50.3) 324 (40.9) 58 (7.3) 12 (1.5)	<0.001
**Complicated Cystitis** **^1^**	110 (27.7)	121 (30.6)	231 (29.1)	0.38
**Complicating factors** **Symptom duration > 7 d** **Recent antibiotic use** **Uncontrolled diabetes** **Urinary tract abnormality** **Renal Transplant** **Catheter use**	58 (14.6) 41 (10.3) 15 (3.8) 1 (0.3) 1 (0.3) 8 (2.0)	71 (17.9) 44 (11.1) 20 (5.1) 0 0 7 (1.8)	129 (16.3) 85 (10.7) 35 (4.4) 1 (0.1) 1 (0.1) 15 (1.9)	0.21 0.72 0.38 0.32 0.32 0.80
**Symptoms** **Dysuria** **Urgency** **Frequency** **None**	334 (84.1) 230 (57.9) 309 (77.8) 8 (2.0)	340 (85.9) 233 (58.8) 314 (79.3) 4 (1.0)	674 (85.0) 463 (58.4) 623 (78.6) 12 (1.5)	0.50 0.80 0.62 0.25
**Culture Ordered**	260 (65.5)	234 (59.1)	494 (62.3)	0.063
**Organism grown**	228/260 (87.7)	187/234 (79.9)	415/494 (84.0)	0.019
**Organism susceptible to empiric treatment** **^2^**	201/228 (88.2)	173/187 (92.5)	374/415 (90.1)	0.14
**Organism(s) isolated** ***Escherichia coli*** ***Klebsiella pneumoniae*** ***Enterococcus faecalis*** ***Enterobacter cloacae*** ***Pseudomonas aeruginosa*** ***Citrobacter freundii*** ***Citrobacter koseri*** ***Proteus mirabilis*** **Mixed microbiota**	127 (55.7) 12 (5.3) 8 (3.5) 2 (0.9) 3 (1.3) 1 (0.4) 3 (1.3) 6 (2.6) 79 (34.6)	108 (57.8) 14 (7.5) 5 (2.7) 3 (1.6) 0 3 (1.6) 3 (1.6) 2 (1.1) 50 (26.7)	235 (56.6) 26 (6.3) 13 (3.1) 5 (1.2) 3 (0.7) 4 (1.0) 6 (1.4) 8 (1.9) 129 (31.1)	0.67 0.35 0.63 0.50 0.12 0.23 0.81 0.25 0.083

Abbreviations: AA, African American; AI, American Indian; SMX/TMP, Sulfamethoxazole**/**trimethoprim; MD, medical doctor; DO, doctor of osteopathy; APP, advanced practice provider.

1 Complicating factors included: male sex, age greater than 65, pregnant, symptom duration greater than 7 days, recent antimicrobial use (30 d), poorly controlled diabetes (A1c >7%), history of infection with multidrug-resistant organism(s), urinary obstruction or anatomic abnormality of urinary tract, current indwelling ureteral stent, nephrostomy tube, urinary diversion, or renal transplant.

2 Of the 27 cultures in the panel group resistant to empiric therapy, 9 were due to resistant *Escherichia coli* isolates. SMX/TMP has a known concern with increasing *E. coli* resistance,^29^ for which a disclaimer exists in the order panel, and was prescribed in 4 of the 9 encounters. Other common isolates responsible for resistance to empiric therapy in the panel use group included *Klebsiella pneumoniae* (6), *Klebsiella aerogenes* (4), *Enterococcus faecalis* (3), and *Pseudomonas aeruginosa* (3).

Antimicrobial prescribing guideline concordance was higher in the panel use cohort as compared with non-panel cohort (79.3% vs 64.9%, *P* < 0.001) (Table 2). Additionally, a statistically significant difference in antimicrobial duration was observed between the panel and no panel cohorts, despite both groups having median of 5 days and IQR of 5–7 days. This was likely secondary to a higher proportion of 3- and 5-day regimens utilized in the panel cohort compared with the non-panel cohort (Table 2). Inappropriate duration was the most common reason for guideline non-concordance in both panel use (87.8%) and non-panel use (84.9%) cohorts. Nitrofurantoin was the most utilized antibiotic in both the panel use (70%) and non-panel use (67.7%) cohorts. Higher use of sulfamethoxazole/trimethoprim (11.6% vs 6.6%; *P* = 0.014) and lower use of cefdinir (10.1% vs 14.9%; *P* = 0.04) were observed in the panel cohort as compared with non-panel cohort, respectively. Antibiotic change was observed in 13.9% of the panel cohort and 12.1% of the non-panel cohort (*P* = 0.47) (Table 2). Overall, there was no difference in UTI-related repeat healthcare contact (13.4% vs 11.1%, *P* = 0.34) between groups.

**Table 2.** Primary and secondary outcomes

Outcome (N, %)	Panel (N = 397)	No Panel (N = 396)	Total (793)	P-Value
**Guideline Concordance**	315 (79.3)	257 (64.9)	572 (72.1)	<0.001
**Duration (median, IQR)**	5 (5,7)	5 (5,7)	5 (5,7)	0.049
**Reason for non-concordance** **Selection** **Dose/Frequency** **Duration (in days)**	17/82 (20.7) 7/82 (8.5) 72/82 (87.8)	40/139 (28.8) 25/139 (18.1) 118/139 (84.9)	57/221 (25.8) 32/221 (14.5) 190/221 (86)	0.19 0.054 0.55
**Antibiotic Utilization** **Nitrofurantoin** **SMX/TMP** **Cephalexin** **Fosfomycin** **Cefdinir** **Cefadroxil** **Ciprofloxacin** **Levofloxacin** **Amoxicillin** **Amoxicillin/Clavulanate**	278 (70) 46 (11.6) 21 (5.3) 0 40 (10.1) 2 (0.5) 9 (2.3) 1 (0.3) 0 0	268 (67.7) 26 (6.6) 15 (3.8) 1 (0.3) 59 (14.9) 1 (0.3) 19 (4.8) 3 (0.8) 3 (0.8) 2 (0.5)	546 (68.9) 72 (9.1) 36 (4.5) 1 (0.1) 99 (12.5) 3 (0.4) 28 (3.5) 4 (0.5) 3 (0.4) 2 (0.3)	0.48 0.014 0.31 0.32 0.040 0.56 0.054 0.31 0.082 0.16
**Antibiotic change** **^1^**	55 (13.9)	48 (12.1)	103 (13)	0.47
**Repeat Healthcare contact**	53 (13.4)	44 (11.1)	97 (12.2)	0.34

Abbreviations: SMX/TMP, Sulfamethoxazole/trimethoprim.

1 Of 103 encounters that saw a change in antibiotic, 37 (6% (24) vs 3.3% (13) panel, non-panel, respectively) were due to proven microbiological resistance on culture, 8 (4 in each group) were due to adverse effects, and 61 (7.3% vs 8.1%) were due to lack of symptom improvement.

In the multivariable model, use of the order panel (OR, 2.51 [95% CI, 1.71–3.70]; *P* < 0.001) and telemedicine encounters (compared to in-person visits, OR, 10.66 [95% CI, 4.07–27.93]; *P* < 0.001) were associated with guideline-concordant prescribing. In contrast, reduced kidney function, age 35–64, and patients with complicated cystitis were associated with decreased guideline concordance (Table 3).

**Table 3.** Multivariable analysis for guideline concordance

Variable	Odds Ratio (95% CI)	P-value
**Panel (Used vs Not used)**	2.51 (1.71–3.70)	<0.001
**Age (in years)** **^1^** **18–34** **35–64** **65+**	Reference 0.57 (0.33–1.0) 1.22 (0.69–2.15)	0.048 0.50
**Sex (Male vs Female)**	0.79 (0.37–1.69)	0.54
**Encounter Type** **In-person** **Telemedicine** **Non-visit care**	Reference 10.66 (4.07–27.93) 0.91 (0.52–1.58)	<0.001 0.74
**Primary Provider Type** **MD/DO** **APP** **Other** **^2^**	Reference 1.07 (0.69–1.65) 1.32 (0.24–7.35)	0.78 0.75
**Beta Lactam allergy (Yes vs No)**	0.78 (0.49–1.23)	0.28
**Sulfamethoxazole/trimethoprim Allergy (Yes vs No)**	0.72 (0.44–1.16)	0.17
**CrCl** **≥ 30 mL/min** **< 30 mL/min** **Unknown**	Reference 0.10 (0.02–0.42) 1.32 (0.89–1.97)	0.002 0.17
**Complicated Cystitis (Yes vs No)**	0.22 (0.11–0.41)	<0.001
**Symptom duration > 7 d (Yes vs No)**	0.62 (0.32–1.19)	0.15
**Recent antibiotic use (Yes vs No)**	0.53 (0.26–1.06)	0.071

Abbreviations: MD, medical doctor; DO, doctor of osteopathy; APP, advanced practice provider; CrCl, creatinine clearance.

*Odds ratio (OR) >1 indicates more likely to meet guideline concordance, if accompanied by statistically significant P-value

1 Treated as categorical variable given non-linear relationship between age and guideline concordance.

2 Other providers include registered nurses and internal resource pool utilizing institutional protocols.

## Discussion

We sought to retrospectively compare the concordance of antimicrobial prescribing with institutional guidelines for cystitis between encounters with and without use of a pre-populated order panel. A significantly higher rate of prescribing concordance with institutional guidelines was seen among encounters with panel use compared to those without. Furthermore, no statistically significant difference in 14-day repeat healthcare contact for UTI-related indications was observed.

In the inpatient setting, CDS at the time of prescribing has been shown to positively impact antibiotic prescribing by reducing use of broad-spectrum antibiotics antibiotic-associated costs. However, studies looking at the use of CDS in the ambulatory setting are sparse. In a study aiming to optimize UTI treatment, Eudaley et al. found that use of a CDS tool facilitating accurate diagnosis and guideline-concordant antibiotic prescribing, demonstrated a 31% improvement in nitrofurantoin use and a 32% increase in guideline-adherent antibiotic durations.^16^ Other studies have identified significant improvements in diagnostic accuracy (eg, UTI syndromes vs ASB), antibiotic agent selection, and duration after implementation of CDS and provider education.^17,18^ Additionally, embedding local guidelines and treatment algorithms within CDS has been shown to improve tool utilization by prescribers, eliminating the need to navigate outside of the medical record.^19^ Our results align with existing literature highlighting the efficacy of CDS strategies in optimizing antibiotic prescribing.

Among encounters that were non-adherent to institutional guidelines, the most common reason in both groups was inappropriate duration of treatment. This included both inappropriately long and short durations. In the era of “shorter is better” for antibiotic durations, many studies have shown similar rates of clinical efficacy among short (3–5 d) courses as compared with longer (7–14 d) courses, with less adverse effects among the shorter durations.^20–23^ Our results effectively demonstrate that opportunities remain for broader adoption of “less is more,” however, providers may also be conversely shortening pre-populated durations in cases where longer durations of therapy may be justified, possibly resulting from provider assessment of symptom severity, diagnostic uncertainty, or patient expectations. Further studies are warranted regarding validity of some complicating factors (eg, male sex or age >65) and optimal durations of therapy for complicated cystitis, especially given heterogeneity observed between current complicating features.

Higher utilization of trimethoprim/sulfamethoxazole (Table 2) was observed amongst the panel use cohort despite regional variability in trimethoprim/sulfamethoxazole susceptibility in *Escherichia coli* isolates within our enterprise. We hypothesize this increase may be attributed to a panel-based recommendation to verify local antibiogram susceptibilities prior to use along with a direct hyperlink to regional antibiogram data.

Indiscriminate ordering of urinalysis and/or urine cultures in the absence of urinary symptoms can contribute to antibiotic overuse.^24–26^ Neither the 2011 Infectious Diseases Society of America guidelines nor the European guidelines for managing UTIs recommend urine cultures for uncomplicated cystitis.^27,28^ Our order panel recommends urine cultures be considered in uncomplicated cystitis but recommends obtainment for all complicated cystitis encounters. In our study, cultures were ordered in a large proportion of patients (62.3%), though only 29.1% of encounters were complicated cystitis. Furthermore, though not statistically significant, a numerically higher rate of culture obtainment was observed in visits from panel use cohorts highlighting opportunity for diagnostic stewardship.

The multivariable model accounted for variables thought *a priori* to be related to inappropriate prescribing. Telemedicine encounters were significantly more likely to meet guideline concordance, which we hypothesize to be driven by using a strict, systematic telemedicine protocol during which direct questions are asked to obtain information. Questions from this protocol align with many of the recommendations made in the panel, thereby minimizing the chance of missing key data necessary to choose a guideline-concordant regimen. Poor renal function (CrCl less than 30 mL/min), though represented with limited patients, was also a driver of non-concordance with guideline recommendations, which we hypothesize was due to the use of nitrofurantoin, the panel’s first-line recommendation, being contraindicated. Prescribers would then be prompted to choose from sulfamethoxazole/trimethoprim, an antibiotic with variability in regional susceptibilities for uropathogens, or alternatives such as beta-lactams. Complicated cystitis encounters were significantly less likely to receive guideline-adherent regimens, highlighting the need for potential future intervention.

Our study is not without several limitations, the most noteworthy being its retrospective design, thereby limiting our ability to attribute a cause-and-effect relationship or control for unidentified confounders. Providers were exposed to education surrounding appropriate antimicrobial use in the management of UTI in conjunction with education surrounding the availability of the panel. We are unable to ascertain if non-panel prescribing was directly impacted by this education, thus improving initial concordance rates despite panel nonuse and lessening the concordance gap between cohorts. Our initial data model relied on accurate encounter-level diagnostic code selection; however, all charts were reviewed for presence of documented pyelonephritis symptoms and excluded if present. Lastly, repeat UTI-related healthcare contact was defined as healthcare contact for a urinary indication within 14 days of completing antibiotic regimen for UTI symptoms, however, potential for repeat healthcare contact outside of the Mayo Enterprise may have contributed to underestimation of repeat contact rates.

## Conclusion

Implementation of an evidence-based order panel for ambulatory UTI syndromes was associated with improved concordance of antibiotic prescribing with institutional guidelines in encounters for cystitis, without negatively impacting repeat UTI-related healthcare contact. Our findings contribute to this growing body of evidence illustrating that development and adoption of order panels may lead to meaningful improvements in antibiotic prescribing in the ambulatory setting.

## Supporting information

Neumann et al. supplementary material Neumann et al. supplementary material

## Supplementary material

For supplementary material accompanying this paper visit http://doi.org/10.1017/ash.2025.62. click here to view supplementary material
