# Supplementary material for: Impact of an evidence-based order panel on antibiotic prescribing in ambulatory patients with cystitis
Source: Antimicrob Steward Healthc Epidemiol. 2025 Apr 21;5(1):e96. doi: 10.1017/ash.2025.62 (PMC12022930; doi:10.1017/ash.2025.62)
Supplement: Neumann et al. supplementary material [file S2732494X25000622sup001.docx]

**Supplementary Appendix:** Impact of an Evidence-Based Order Panel on Antibiotic Prescribing in Ambulatory Patients with Cystitis

**EZ ID Urinary Order Panel (Adult)**

*Guidance text: Symptoms of cystitis include a combination of dysuria, increased urinary frequency, and increased urgency. In addition to symptoms of cystitis (e.g., dysuria, frequency, urgency, etc.), patients with pyelonephritis may present with costovertebral angle tenderness (i.e., flank pain) and/or fever.*

**Quick links:**

AskMayoExpert (AME) [hyperlink]

Antimicrobial Quick Guide [hyperlink]

Viral Rx Pad [hyperlink]

**Patients with lower urinary symptoms ONLY (i.e., cystitis)**

- *Guidance text: Cystitis is considered* ***complicated*** *if one or more of the following is present: male, age>65, pregnant, symptom duration > one week, recent antimicrobial use, poorly controlled diabetes, history of infection with multidrug-resistant organism(s), urinary obstruction or anatomic abnormality of urinary tract, current indwelling stent, nephrostomy tube, urinary diversion, or renal transplant.*
- *Guidance text:* For symptomatic patients with indwelling catheters, remove or replace catheter.
- Laboratory testing for cystitis
  - *Guidance text: Urinalysis, urine culture, and/or dipstick can be considered in patients with uncomplicated cystitis but is recommended for ALL patients with complicated cystitis.*
  - Bacterial culture, Aerobic and susceptibility, Urine
  - Urinalysis with microscopic
  - Dipstick, POCT, urine
- Treatment of cystitis
  - *Guidance text: Avoid empiric cephalexin or sulfamethoxazole/trimethoprim in regions where urinary E. coli susceptibility rates are < 80%. See antibiogram (link above). Use caution with sulfamethoxazole/trimethoprim in the elderly or those with renal impairment given risk for hyperkalemia.*
  - *Guidance text: This order panel is not intended to guide treatment of prostatitis. See AME for treatment recommendations.*
    - AME - Prostatitis [hyperlink]
  - **Uncomplicated cystitis**
    - Antimicrobial Therapy
      - Preferred first line
        - *Guidance text: Nitrofurantoin should be avoided in patients with CrCl <30 ml/min.*
        - Nitrofurantoin monohydrate 100 mg PO BID x 5 days; Quantity 10; Refill: 0
        - Sulfamethoxazole/Trimethoprim 800/160 mg PO BID x 3 days; Quantity 6; Refill: 0
      - Alternatives with no beta-lactam allergy
        - Cephalexin 500 mg PO BID x 5 days; Quantity 10; Refill: 0
        - Cefdinir 300 mg PO BID x 5 days; Quantity 10; Refill 0
      - Alternative with allergy to cephalosporins
      - *Guidance text: Fosfomycin is expensive and may require prior authorization.*
      - Fosfomycin 3 g PO once; Quantity 1; Refill 0
    - Symptomatic management
      - *Guidance text: Phenazopyridine use is optional and should only be used in combination with an antimicrobial. Prescription insurance coverage of phenazopyridine may vary. Over the counter formulations are available.*
      - Phenazopyridine 200 mg PO TID x 2 days; Quantity 6; Refill: 0; Order comment: If not covered by insurance, please suggest over the counter phenazopyridine.
  - **Complicated cystitis (non-pregnant patient)**
    - *Guidance text: All doses below assume normal renal function. Renal dose adjustments may be necessary for some agents.*
    - Antimicrobial Therapy
      - Preferred first line
        - *Guidance text: Nitrofurantoin should be avoided in patients with CrCl <30 ml/min.*
        - Nitrofurantoin monohydrate 100 mg PO BID x 7 days; Quantity 14; Refill: 0
        - Sulfamethoxazole/Trimethoprim 800/160 mg PO BID x 7 days; Quantity 14; Refill: 0
      - Alternatives with no beta-lactam allergy
        - Cephalexin 500 mg PO BID x 7 days; Quantity 14; Refill: 0
        - Cefdinir 300 mg PO BID x 7 days; Quantity 14; Refill 0
      - Alternative with allergy to cephalosporins
      - *Guidance text: Avoid fluoroquinolones due to adverse effect profile and increasing resistance in common urinary pathogens. Preserve use for more serious infections with proven susceptibility.*
      - *Guidance text: Fosfomycin is expensive and may require prior authorization.*
      - Ciprofloxacin 250 mg PO BID x 5 days; Quantity: 10; Refill: 0
      - Fosfomycin 3 g PO once; Quantity 1; Refill 0
    - Symptomatic management
      - *Guidance text: Phenazopyridine use is optional and should only be used in combination with an antimicrobial. Prescription insurance coverage of phenazopyridine may vary. Over the counter formulations are available.*
      - Phenazopyridine 200 mg PO TID x 2 days; Quantity 6; Refill: 0; Order comment: If not covered by insurance, please suggest over the counter phenazopyridine.
  - **Cystitis in pregnancy**
    - Gestation: <13 weeks
      - Preferred first line
        - Cephalexin 500 mg PO QID x 7 days; Quantity 28; Refill: 0
        - Cefdinir 300 mg PO BID x 7 days; Quantity 14; Refill 0
      - Alternatives
        - *Guidance text: Nitrofurantoin may be associated with congenital anomalies if used in the first trimester (mixed data); but reasonable to use if no appropriate alternative. Used with caution at term or when labor is imminent due to caution for hemolytic anemia in the neonate, especially in patients with known G6PD deficiency.*
        - *Guidance text: Fosfomycin is expensive and may require prior authorization.*
        - Nitrofurantoin monohydrate 100 mg PO BID x 7 days; Quantity 14; Refill 0
        - Fosfomycin 3 g PO every 3 days; Quantity 2; Refill 0
    - Gestation: ≥13 weeks to <37 weeks
      - Preferred first line
        - *Guidance text: Nitrofurantoin is contraindicated for pregnant patients at term (i.e., after week 37 of pregnancy) given potential for hemolytic anemia of the neonate.*
        - Nitrofurantoin monohydrate 100 mg PO BID x 7 days; Quantity 14; Refill 0
      - Alternatives
        - Cephalexin 500 mg PO QID x 7 days; Quantity 28; Refill: 0
        - Cefdinir 300 mg PO BID x 7 days; Quantity 14; Refill 0
    - Gestation: >37 weeks
      - Preferred first line
        - Cephalexin 500 mg PO QID x 7 days; Quantity 28; Refill: 0
        - Cefdinir 300 mg PO BID x 7 days; Quantity 14; Refill 0
      - Alternatives
        - *Guidance text: Fosfomycin is expensive and may require prior authorization.*
        - Fosfomycin 3 g PO every 3 days; Quantity 2; Refill 0
  - **Recurrent cystitis**
    - *Guidance text: Recurrent cystitis refers to the presence of ≥2 episodes of UTI in 6 months or ≥3 episodes in 1 year. In patients with recurrent cystitis, referral to urology and/or infectious diseases may be considered.*
    - *Guidance text: Long term antimicrobial prophylaxis is not recommended.*
    - *Guidance text: All doses below assume normal renal function. Renal dose adjustments may be necessary for some agents.*
    - Antimicrobial Therapy
      - Preferred first line
        - *Guidance text: Nitrofurantoin should be avoided in patients with CrCl <30 ml/min.*
        - Nitrofurantoin monohydrate 100 mg PO BID x 7 days; Quantity 14; Refill 0
        - Sulfamethoxazole/Trimethoprim 800/160 mg PO BID x 7 days; Quantity 14; Refill: 0
      - Alternatives with no beta-lactam allergy
        - Cephalexin 500 mg PO BID x 7 days; Quantity 14; Refill 0
        - Cefdinir 300 mg PO BID x 7 days; Quantity 14; Refill 0
      - Alternative with allergy to cephalosporins
        - *Guidance text: Avoid fluoroquinolones due to adverse effect profile and increasing resistance in common urinary pathogens. Preserve use for more serious infections with proven susceptibility.*
        - *Guidance text: Fosfomycin is expensive and may require prior authorization.*
        - Ciprofloxacin 250 mg PO BID x 5 days; Quantity: 10; Refill: 0
        - Fosfomycin 3 g PO once; Quantity 1; Refill 0
    - Symptomatic management
      - *Guidance text: Phenazopyridine use is optional and should only be used in combination with an antimicrobial.*
      - *Guidance text: For women with atrophic vaginitis, estrogens my help prevent recurrent cystitis.*
      - Phenazopyridine 200 mg PO TID x 2 days; Quantity 6; Refill: 0
      - Estrogens
        - Estrogens, conjugated 1g (0.625 mg/gram) vaginal cream – Insert 0.5 g into the vagina at bedtime for 14 days, then 0.5 g two times weekly; Quantity 30 g; Refill: 2
        - Estradiol 0.1 mg/g (0.01%) vaginal cream – Insert 0.5 g into the vagina at bedtime for 14 days, then 0.5 g two times weekly; Quantity 42.5 g; Refill: 2

**Patient with upper urinary symptoms (i.e., pyelonephritis)**

*Guidance text: Indications for admission: Pregnancy, hemodynamic instability, sepsis, urinary retention/obstruction, need for IV therapy, or bacteremia.*

AME – UTI pyelonephritis imaging recommendations [hyperlink]

- Laboratory testing for pyelonephritis
  - *Guidance text: Urine culture and susceptibility should be ordered on all patients with suspected pyelonephritis to guide antimicrobial therapy.*
  - Urinalysis with Microscopic: Urine, Midstream
  - Bacterial culture, Aerobic + Susceptibility, Urine
  - Bacteria/Candida Culture, Blood
- Treatment of pyelonephritis
  - *Guidance text: Most pyelonephritis may be treated with a 7-day course; consider extending up to 14 days in patients who are immunocompromised or have complex urologic anatomy.*
  - *Guidance text: Initial treatment of pyelonephritis may require the use of IV antibiotics. Antimicrobial therapy should be guided by cultures and susceptibility data.*
  - *Guidance text: All doses below assume normal renal function. Renal dose adjustment may be necessary for some agents.*
  - Oral Antimicrobials
    - Sulfamethoxazole/Trimethoprim 800/160 mg PO BID x 7 days; Quantity 14; Refill: 0
    - Cefdinir 300 mg PO PO BID x 10 days; Quantity 20; Refill: 0
    - Ciprofloxacin 500 mg PO BID x 7 days; Quantity 14; Refill 0
    - Levofloxacin 750 mg PO QD x 7 days; Quantity 7; Refill 0

Patient is **WITHOUT** symptoms (i.e., asymptomatic bacteriuria)

*Guidance text: Screening of asymptomatic patients should ONLY be considered in patients who are pregnant or prior to urologic procedure.*

- Laboratory screening for asymptomatic bacteriuria
  - *Guidance text: Screening consists of urine bacterial culture and susceptibility testing. Urinalysis is not indicated.*
  - *Guidance text: Foul smelling or cloudy urine is not suggestive of UTI and should not prompt laboratory workup.*
  - Bacterial Culture, Aerobic + Susceptibility, Urine
- Treatment of asymptomatic bacteriuria
  - *Guidance text: Antimicrobial therapy for post-renal transplant patients or those undergoing urologic procedures should be guided by transplant nephrology or urology, respectively.*
  - *Guidance text: All doses below assume normal renal function. Renal dose adjustment may be necessary for some agents.*
  - Pregnant
    - *Guidance text: Antimicrobial therapy should be guided by screening culture results.*
    - Gestation: <13 weeks
      - Preferred first line
        - Cephalexin 500 mg PO QID x 5 days; Quantity 20; Refill: 0
        - Cefdinir 300 mg PO BID x 5 days; Quantity 10; Refill 0
      - Alternatives
        - *Guidance text: Nitrofurantoin may be associated with congenital anomalies if used in the first trimester (mixed data); but reasonable to use if no appropriate alternative. Used with caution at term or when labor is imminent due to caution for hemolytic anemia in the neonate, especially in patients with known G6PD deficiency.*
        - *Guidance text: Fosfomycin is expensive and may require prior authorization.*
        - Nitrofurantoin monohydrate 100 mg PO BID x 5 days; Quantity 10; Refill 0
        - Fosfomycin 3 g PO x 1 dose; Quantity 1; Refill 0
    - Gestation: ≥13 weeks to <37 weeks
      - Preferred first line
        - *Guidance text: Nitrofurantoin is contraindicated for pregnant patients at term (i.e., after week 37 of pregnancy) given potential for hemolytic anemia of the neonate.*
        - Nitrofurantoin monohydrate 100 mg PO BID x 5 days; Quantity 10; Refill 0
      - Alternatives
        - Cephalexin 500 mg PO QID x 5 days; Quantity 20; Refill: 0
        - Cefdinir 300 mg PO BID x 5 days; Quantity 10; Refill 0
    - Gestation: >37 weeks
      - Preferred first line
        - Cephalexin 500 mg PO QID x 5 days; Quantity 20; Refill: 0
        - Cefdinir 300 mg PO BID x 5 days; Quantity 10; Refill 0
      - Alternatives
        - *Guidance text: Fosfomycin is expensive and may require prior authorization.*
        - Fosfomycin 3 g PO every 3 days; Quantity 2; Refill 0

**EZ ID Urinary Order Panel (Pediatric)**

*Guidance text: This panel is intended for use in non-pregnant patients < 18 years of age.*

*Guidance text: A urinary tract infection (UTI) is defined as growth of >1,000 CFU/mL of a single organism from the urine of a patient with compatible symptoms and supportive urinalysis (>10 WBC/HPF, positive gram stain, positive nitrite test, OR positive leukocyte esterase test). Confirmation of UTI diagnosis requires a positive urine culture.*

**Quick links:**

AskMayoExpert (AME) [hyperlink]

Antimicrobial Quick Guide [hyperlink]

Viral Rx Pad [hyperlink]

- Laboratory
  - *Guidance text:* *Urine specimens should be obtained via catheterization from children who are not toilet trained, or who are toilet trained but clean-catch specimen is suggestive of contamination. See AME – UTI (child) link for additional testing guidance.*
  - Urinalysis with Microscopic if Indicated
  - Bacterial Culture, Aerobic and Susceptibility, Urine
- Medications
  - *Guidance text: When available, antimicrobial selection should be guided by culture and sensitivity data. If culture is negative, antibiotics started empirically should be stopped.*
  - **Cystitis**
    - Children > 2 years of age
      - First line/Primary/Preferred
        - Nitrofurantoin

*Guidance text: Recommended nitrofurantoin (FURADANTIN) suspension dosing 6 mg/kg/day (max dose 400 mg/day)*

*Guidance text: Age > 11 years OR weight > 40 kg: Nitrofurantoin monohydrate (MACROBID) 100 mg BID*

*Guidance text: Choose preferred formulation.*

Nitrofurantoin (FURADANTIN) 25mg/5 ml suspension – Take 6 mg /kg/day PO QID x 7 days.

- - - - - Cephalexin

*Guidance text: Recommended dosing 45 mg/kg/day (max dose 1500 mg/day): Choose preferred formulation.*

Cephalexin 250 mg/5ml suspension –Take 45 mg/kg/day PO every 8 hours x 7 days.

Cephalexin 250 mg capsule – Take 45 mg/kg/day PO every 8 hours x 7 days.

- - - - Gram stain results as gram-positive cocci (concern for Enterococcus)
        - Amoxicillin

*Guidance text: Recommended dosing 30 mg/kg/day (max dose 1500 mg/day): Choose preferred formulation.*

Amoxicillin 400 mg/5ml suspension – Take 30 mg/kg/day PO every 8 hours x 7 days.

Amoxicillin 500 mg capsule - Take 30 mg/kg/day PO every 8 hours x 7 days.

- - - - Alternatives
        - Sulfamethoxazole/Trimethoprim

*Guidance text: Recommended sulfamethoxazole-trimethoprim dosing 10 mg/kg/day of trimethoprim (max dose 320 mg/day). Choose preferred formulation.*

Sulfamethoxazole/Trimethoprim 200-40/5 ml suspension – Take 10 mg/kg/day of trimethoprim PO every 12 hours x 7 days.

Sulfamethoxazole/Trimethoprim 400-80 mg tablet – Take 10 mg/kg/day of trimethoprim PO every 12 hours x 7 days.

Sulfamethoxazole/Trimethoprim 800-160 mg tablet – Take 10 mg/kg/day of trimethoprim PO every 12 hours x 7 days.

- - - - - Cefdinir

*Guidance text: Recommended dosing 7 mg/kg/dose (max dose 300 mg). Choose preferred formulation.*

Cefdinir 250 mg/5ml suspension – Take 7 mg/kg PO BID x 7 days.

Cefdinir 300 mg capsule – Take 7 mg/kg PO BID x 7 days.

- - Pyelonephritis
    - Children > 2 years of age
      - *Guidance text: An afebrile, well appearing child who is not vomiting is a candidate for oral antibiotics.*
      - First line/Primary/Preferred
        - Cephalexin

*Guidance text: Recommended dosing 45 mg/kg/day (max dose 1500 mg/day): Choose preferred formulation.*

Cephalexin 250 mg/5ml suspension –Take 45 mg/kg/day PO every 8 hours x 10 days.

Cephalexin 250 mg capsule – Take 45 mg/kg/day PO every 8 hours x 10 days.

- - - - History of Enterococcus UTI or if the gram stain shows gram-positive cocci
        - Amoxicillin

*Guidance text: Recommended dosing 30 mg/kg/day (max dose 1500 mg/day): Choose preferred formulation.*

Amoxicillin 400 mg/5ml suspension – Take 30 mg/kg/day PO every 8 hours x 10 days.

Amoxicillin 500 mg capsule - Take 30 mg/kg/day PO every 8 hours x 10 days.

- - - - Alternatives
        - Sulfamethoxazole/Trimethoprim

*Guidance text: Recommended sulfamethoxazole-trimethoprim dosing 10 mg/kg/day of trimethoprim (max dose 320 mg/day). Choose preferred formulation.*

Sulfamethoxazole/Trimethoprim 200-40/5 ml suspension – Take 10 mg/kg/day of trimethoprim PO every 12 hours x 10 days.

Sulfamethoxazole/Trimethoprim 400-80 mg tablet – Take 10 mg/kg/day of trimethoprim PO every 12 hours x 10 days.

Sulfamethoxazole/Trimethoprim 800-160 mg tablet – Take 10 mg/kg/day of trimethoprim every 12 hours x 10 days.

- - - - - Cefdinir

*Guidance text: Recommended dosing 7 mg/kg/dose (max dose 300 mg). Choose preferred formulation.*

Cefdinir 250 mg/5ml suspension – Take 7 mg/kg PO BID x 10 days.

Cefdinir 300 mg capsule – Take 7 mg/kg PO BID x 10 days.
